# NT-proBNP in the very old population: reference values and heart failure specificity from the AugUR-study

**DOI:** 10.1007/s00392-025-02785-3

**Published:** 2025-10-27

**Authors:** Maria A. Heinrich, Klaus J. Stark, Martina E. Zimmermann, Janina M. Herold, Caroline Brandl, Alexander D. Schober, Ralph Burkhardt, Lars S. Maier, Andreas Luchner, Iris M. Heid, Alexander Dietl

**Affiliations:** 1 Department of Internal Medicine II, University Hospital Regensburg, Regensburg, Germany; 2 Department of Genetic Epidemiology, University of Regensburg, Regensburg, Germany; 3 Department of Ophthalmology, University Hospital Regensburg, Regensburg, Germany; 4 Institute of Clinical Chemistry and Laboratory Medicine, University Hospital Regensburg, Regensburg, Germany; 5 Department of Cardiology, Krankenhaus Barmherzige Brueder Regensburg, Regensburg, Germany

**Keywords:** NT-proBNP, Elderly, Population-based, Specificity, Reference value

## Abstract

**Background:**

High NT-proBNP is an established marker of heart failure. However, old individuals have elevated values even when cardiovascularly healthy. Reference values remain scarce.

**Objectives:**

We sought to provide reference values of NT-proBNP for the healthy old and very old population and the proportion above the AHA- and ESC-recommended rule-out cut-off for heart failure diagnosis in a non-acute setting (≥ 125 pg/mL).

**Methods:**

We analyzed cross-sectional data from the population-based AugUR study of inhabitants ≥ 70 years of age. NT-proBNP was determined in serum. Cardiac morphology and function were evaluated by echocardiography.

**Results:**

A total of 2304 subjects aged 70 to 95 years were analyzed (52% women; 35% ≥ 80 years). Median NT-proBNP increased by age group (70–74/75–79/80–84/ ≥ 85 years: 153.0/176.9/228.5/395.0 and 116.1/171.5/217.6/437.0 pg/mL for women or men, respectively); 68% had NT-proBNP ≥ 125 pg/mL. In a subgroup free of cardiovascular risk factors, renal failure, coronary artery disease, and atrial fibrillation (*n* = 685), 50% (70–79 years) and 70% (≥ 80 years) had NT-proBNP ≥ 125 pg/mL; 97.5% quantiles were considerably higher than 125 pg/mL (70–79 years: 559.5 or 424.5 for women or men; 80–95 years: 809.2 or 722.0 pg/mL for women or men). When further restricting to normal left ventricular mass, E/e′ ratio, and ejection fraction (*n* = 65), 53% of subjects (70–79 years) or 50% (≥ 80 years) had NT-proBNP ≥ 125 pg/mL.

**Conclusions:**

NT-proBNP levels continue to rise in the very old population. More than 50% of healthy subjects aged 70 years and older have NT-proBNP values above the decision-making cut-off at 125 pg/mL, potentially causing unnecessary further diagnostics and referrals.

**Graphical Abstract:**

**Figure:**
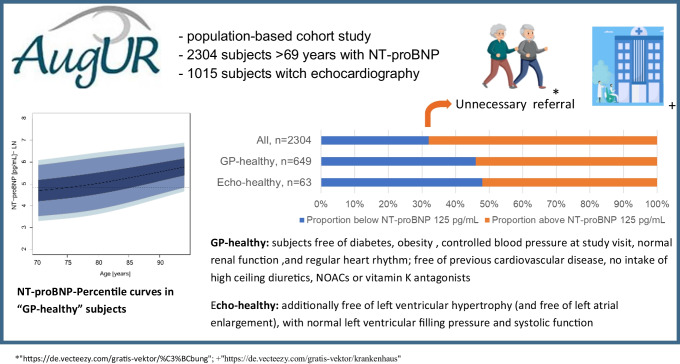

**Supplementary Information:**

The online version contains supplementary material available at 10.1007/s00392-025-02785-3.

## Introduction

The N-terminal cleavage product of B-type natriuretic peptide (NT-proBNP) is a marker of heart failure, as NT-proBNP values are associated with left ventricular diastolic and systolic dysfunction [1, 2]. General practitioners, emergency physicians, and cardiologists use NT-proBNP levels for heart failure screening and diagnosis.

NT-proBNP levels rise with age and are higher in women than in men [3]. However, the current guidelines of the American Heart Association (AHA) and the European Society of Cardiology (ESC) recommend an ambulatory rule-out cut-off value of 125 pg/mL independent of sex and age due to limited data in higher age groups [2, 4]. Thus, sensitivity is excellent and only a few patients are missed, but specificity is low and upper reference limits are supposed to be substantially higher in older individuals [5–7]. Using an age- and sex-independent cut-off value leads to a large number (about 74% in Belgium) of unnecessary cardiologic referrals in patients aged 80 years and older [8]. The need for age-dependent cut-off values is further intensified, as about 70% of all NT-proBNP measurements are done in older patients [9]. Despite the evident gap of knowledge and clinical need, reports on NT-proBNP distribution in the older population and their healthy subgroups are still scarce [5, 10].

The AugUR study was tailored to the needs of the elderly population, aged ≥ 70 years, living in/around the city of Regensburg, Germany, to further the understanding of risk factors and biomarkers for age-related diseases (Age-related diseases: understanding genetic and non-genetic influences—a study at the University of Regensburg).

To address this gap of NT-proBNP reference data in the old and very old population, we analyzed cross-sectional population-based data from 2304 AugUR participants with valid NT-proBNP measurements. Concurrent with the date of blood draw, information on history of cardiovascular disease, blood pressure, diabetes status, and—for a substudy with 1008 participants—cardiac morphology and function were surveyed.

## Methods

### Study sample

**Fig. 1:**
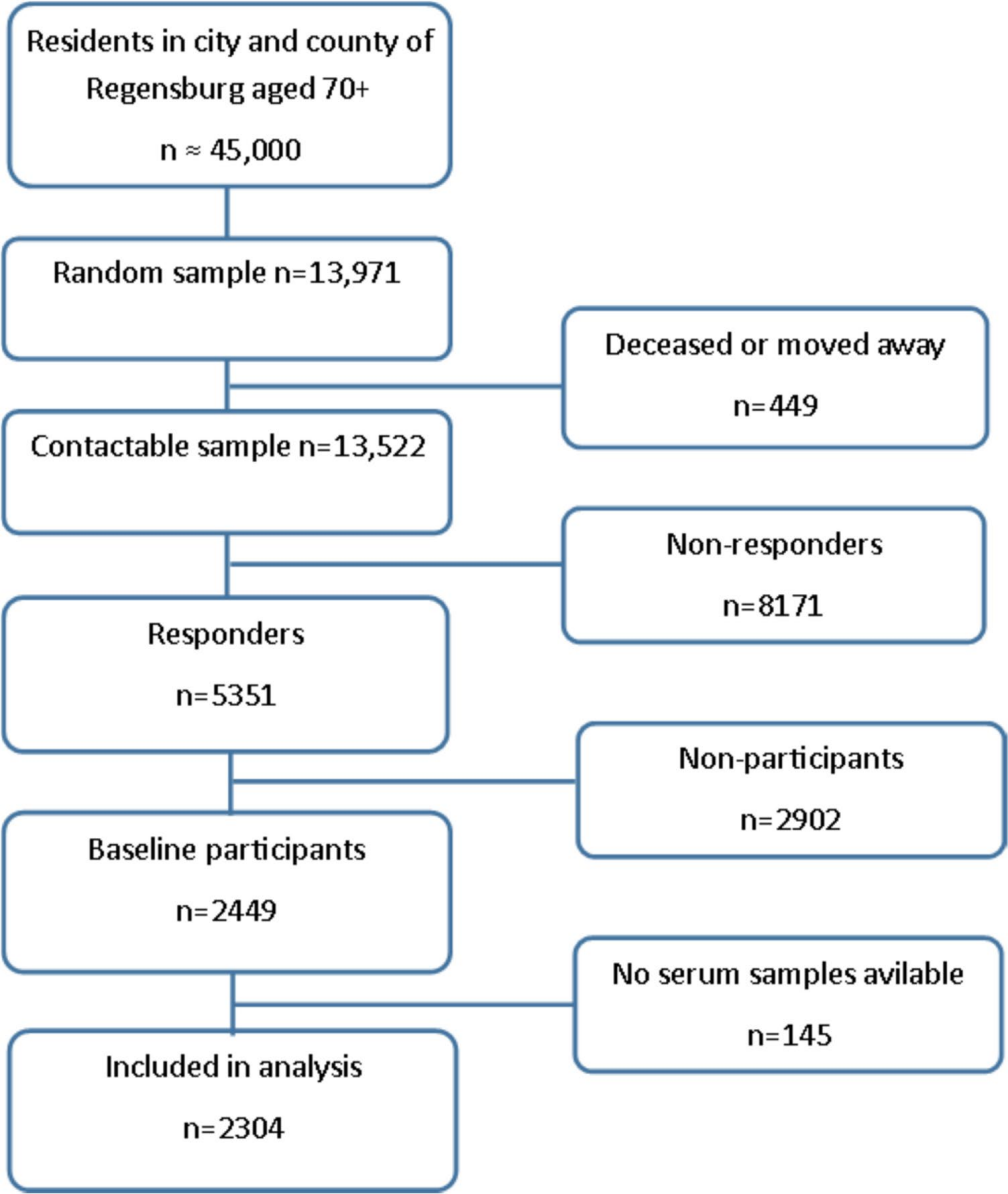
Overview of AugUR study recruitment

The AugUR study is a prospective population-based study, which recruited inhabitants at least 70 years of age in the city of Regensburg, Germany, and nearby counties via random sample from the local registries of residence [11]. The study protocol of the German AugUR study was described previously [12]. In total, 2449 out of 13,522 contacted individuals responded positively (response rate 18.1%) in two independent rounds (*n* = 1133 in AugUR1 2013–2015 and *n* = 1316 in AugUR2 2017–2019) [13]. A total of 2304 participants with available NT-proBNP measurements were included in our analysis (Fig. 1).

### Ethics statement

The study protocol, study procedures, and data protection strategy were all approved by the Ethics Committee of the University of Regensburg, Germany (vote 12–101–0258). All participants who were included gave written consent after being informed about the study. The study was conducted according to the principles expressed in the Declaration of Helsinki.

### Assessment of sociodemographic factors, cardiovascular risk factors, medical history, and blood pressure

Trained staff assessed sociodemographic factors, smoking status, and medical history, including medical regimen and cardiovascular diseases (existence, time of onset, interventions), through a standardized face-to-face interview [14]. Blood pressure and heart rate were measured three times (using an automatic device: Omron M10-IT; Omron Healthcare, Kyoto, Japan), and the average of the second and third measurements was computed. Diabetes mellitus was defined by self-reported diabetes and/or intake of oral antihyperglycemic agents or insulin [15]. Inadequately controlled blood pressure was defined as RR ≥ 160/100 mmHg at the study visit.

### Assessment of cardiac morphology and function by echocardiography

Participants received transthoracic echocardiography following a standardized operating procedure [12]. A commercially available ultrasound unit was used (HP Sonos 5500 with a 2–4 MHz probe; Philips, Eindhoven, The Netherlands). Xcelera R3.2LI V.3.2.1.520–2011 (Philips Medical Systems, Amsterdam, The Netherlands) was employed for post hoc analyses of stored tracings by trained staff according to a standardized protocol [11]. Left atrial volume was calculated by monoplane volumetric measurements in the apical four-chamber view. Normal left atrial volume-index was defined as left atrial volume/body surface area ≤ 34 mL/m^2^. M-mode was used to evaluate left ventricular mass (LVM) in the parasternal long-axis view. LVM was calculated by the Devereux formula. Left atrial volume and LVM were indexed to body surface area (BSA) approximated by DuBois’ formula [16]. A normal LVM/BSA-index was set as ≤ 95 g/m^2^ for women and ≤ 115 g/m^2^ for men. Left ventricular ejection fraction (LVEF) was estimated by the monoplane method of discs (modified Simpson’s rule) in the apical four-chamber view [17, 18]. To estimate diastolic dysfunction in participants with normal LVEF, mitral flow velocities (E′, A) and septal and lateral mitral annular velocity (e′), E/e′ ratio was used [19]. Normal systolic function was defined as LVEF ≥ 50% and normal ventricular filling pressure as E/e′ ratio ≤ 14. The interval between the onset of transmitral inflows was assessed by pulsed-wave Doppler to measure the length of a cardiac cycle. Heart rate was calculated by dividing 1 min by the length of the cardiac cycle. All measurements were repeated three times in regular heart rhythm and 10 times in arrhythmia to reduce random error.

### Blood sampling and natriuretic peptide measurements

Blood sampling and storage was already described in detail [11]. In summary, non-fasting blood samples were taken after at least 5 min resting time. Blood samples were immediately centrifuged and supernatants from serum tubes were stored at − 80 °C. Until final measurement, no freezing or thawing cycles were performed. For NT-proBNP measurement, stored samples were thawn. Laboratory analyses from biobanked samples were performed in compliance with the “Guidelines of the German Medical Association for Quality Assurance of Medical Laboratory Tests” (RiLiBäK) at the Central Laboratory of the University Hospital Regensburg, which is accredited in accordance with the standard DIN EN ISO 15189. Serum samples were stored at − 80 °C for a median time of 4.2 years (minimum 2.0 and maximum 6.7 years).

The measurements were conducted on a cobas e411 (Roche Diagnostics, Rotkreuz, Switzerland). The used NT-proBNP assay has a measurement range from 5 to 35,000 pg/mL, with a lower limit of detection (LoD) of 5 pg/mL and a limit of quantitation (LoQ) of 50 pg/mL. Thereafter, data were exported from SWISSLAB (NEXUS SWISSLAB GmbH, Berlin, Germany) in Excel format and processed with Microsoft Access 2019 Redmond, Washington, USA), SAS V.9.4 (SAS Institute Inc) and SPSS V.29 (IBM Corporation). Renal function was determined with estimated glomerular filtration rate (eGFR) creatinine-based using the CKD-Epi equation [20].

### Statistical methods

SPSS IBM statistics version 29 and R version 4.3.1 were used. Continuous variables are reported as mean and SD or as median with 25th and 75th percentiles. We derived the proportion of individuals above 125 pg/mL, the NT-proBNP cut-off value to exclude chronic heart failure in non-acute patients [2]. We did this in the total group of participants with valid measures of NT-proBNP for our analysis to provide the distribution in a population of old and very old individuals.

To provide reference values in the cardiovascular healthy old and very old, we derived three groups with increasingly strict exclusion to focus on healthy individuals: (1) First, we focused on individuals who had no risk factors for cardiovascular disease. For this, we selected subjects of Subgroup I to be free of diabetes and obesity (BMI < 30 kg/m^2^) and had controlled blood pressure at study visit (< 160/100 mmHg) (subgroup “without cardiovascular risk factors”). (2) Second, we further restricted individuals that would have been deemed cardio-renal healthy after a routine check-up with the GP. Thus, Subgroup II included Subgroup I participants who further had normal renal function (eGFR ≥ 60 mL/min/1.73 m^2^), regular heart rhythm (no arrhythmias during blood pressure measurement or echocardiographic exam), were free of previous cardiovascular disease (self-reported stent, bypass graft, myocardial infarction), and did not take high ceiling diuretics, NOACs, or vitamin K antagonists (subgroup “general practitioner GP-healthy”). (3) Third, we focused on individuals who would have been considered cardiovascularly healthy after echocardiographic examination: Thus, Subgroup III included Subgroup II participants who were further free of left ventricular hypertrophy (left ventricular mass to body surface area ≤ 95 g/m^2^ for women; ≤ 115 g/m^2^ for men) and of left atrial enlargement (left atrial volume/body surface area ≤ 34 mL/m^2^) and had normal left ventricular filling pressure (E/e′-ratio ≤ 14) and normal systolic function (EF ≥ 50%) (subgroup “echo-healthy”).

To derive reference values for NT-proBNP levels in dependency of age and by sex, we focused on subgroup II individuals (“GP-healthy”). We used a generalized additive mixed model for location, scale, and shape (GAMLSS). This approach of distributional regression allowed us to model not only the expected value *µ*_i_ (location) as a function of age but also the variance *σ*_i_ (scale) and skewness (shape), ${\mu }_{\mathrm{i}}={\beta }_{0}+f\left({\mathrm{age}}_{\mathrm{i}}\right)$, ${\sigma }_{\mathrm{i}}= {\mathrm{log}(\delta }_{0}+f\left({\mathrm{age}}_{\mathrm{i}}\right)$, and ${\eta }_{\mathrm{i}}={\alpha }_{0}$ (*i* denoting the *i*th participant and ${\beta }_{0}$, ${\delta }_{0}$, and ${\alpha }_{0}$ the regression parameters. Using the model regression estimates, we derived 2.5th, 25th, 50th, 75th, and 97.5th percentile curves using the *centiles.plot()* function from the *gamlss* R package.

## Results

### Characteristics of the analyzed individuals

Our analyzed data comprised 2304 participants with valid NT-proBNP measurement. Mean age was 78 ± 5 years and ranged from 70 to 95 years; 52% were women. The proportion of male participants with diabetes mellitus and coronary artery disease was higher than that of women (diabetes mellitus 23.2 vs. 18.9%; coronary artery disease 20.4 vs. 6.8%; Table 1). Of the 1008 participants in the echocardiographic substudy, 90.7% (*n* = 885 of 976 with available LVEF) had a normal left ventricular ejection fraction (EF ≥ 50%). Left ventricular mass index (LVMi) and left arterial volume index were normal for 52.8% (*n* = 413 of 782) and 40.8% (*n* = 392 of 961), respectively (Table 1).

**Table 1:** Participant characteristics of the analyzed sample. Shown are mean and SD or proportions (if not indicated otherwise) for the 2304 subjects separately for women and men. Characteristics for the echo-substudy are given for 1008 participants

Characteristic*s*	Women	*n*	Men	*n*
NT-proBNP (pg/mL) median (P25; P75)	195.0 (116.0; 376.0)	1198	180.0 (96.2; 396.0)	1106
Age (years)	78.7 ± 4.9	1198	78.5 ± 5.1	1106
Body mass index (kg/m^2^)	27.6 ± 5.0	1192	27.8 ± 3.9	1100
eGFR (mL/min/1.73m^2^)	67.8 ± 16.7	1191	67.7 ± 16.6	1099
LDL-cholesterol (mg/dL)	147.6 ± 34.6	1196	133.6 ± 34.1	1106
Inadequately controlled blood pressure (*n* (%))	80 (6.7)	1194	93 (8.4)	1104
Diabetes (*n* (%))	226 (18.9)	1197	258 (23.2)	1106
Coronary artery disease (*n* (%))	79 (6.8)	1168	217 (20.4)	1063
Tobacco use (present/past) (*n* (%))	347 (29.1)	1191	678 (61.4)	1102
Regular rhythm (%)	981 (82.2)	1193	783 (71.1)	1101
Heart rate (bpm)	71.1 ± 11.1	1194	68.6 ± 12.3	1104
Echo-substudy				
LAVI (mL/m^2^)	39.1 ± 15.3	426	39.1 ± 15.3	535
LVMi (g/m^2^)	94.1 ± 25.2	365	117.8 ± 32.7	417
E/e′ ratio	11.7 ± 3.9	394	11.2 ± 3.6	483
LVEF (%)	61.9 ± 6.8	440	59.3 ± 8.0	536

*n* with available data

*eGFR* glomerular filtration rate estimated from serum creatinine using the CKD-Epi equation (mL/min/1.73 m^2^); inadequately controlled blood pressure: RR ≥ 160/100 mmHg at the study visit; coronary artery disease: self-reported myocardial infarction, stents, coronary artery bypass grafting; *LAVI* left atrial volume index (left atrial volume/body surface area); *LVMi* the ratio of left ventricular mass to body surface area; E/e′ ratio: mitral valve E velocity divided by mitral annular e′ velocity; *LVEF* left ventricular ejection fraction

### Distribution of NT-proBNP values in old and very old participants

**Fig. 2:**
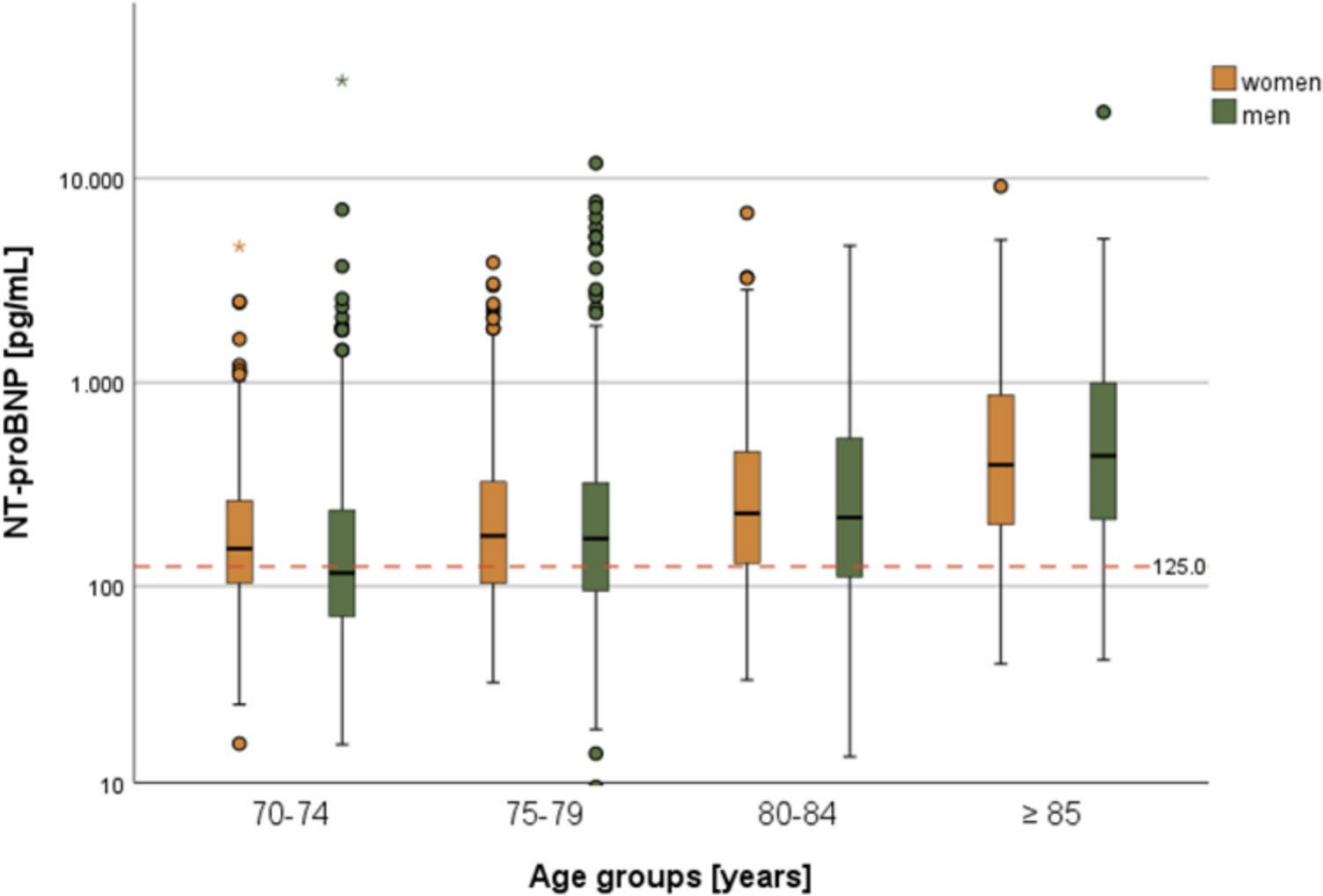
NT-proBNP values stratified for sex and age. Values of NT-proBNP in 2304 participants of the AugUR study. A box represents the lower (25%) and upper (75%) quartiles, with the median as a horizontal line within the box. The *Y*-axis shows NT-proBNP values on a log 10-based scale. Values marked with circles are within the 3rd interquartile range (IQR), and values marked with stars are above the 3rd IQR. Maximum NT-proBNP value: 30,122.0 pg/mL; minimum value: 9.5 pg/mL

**Fig. 3:**
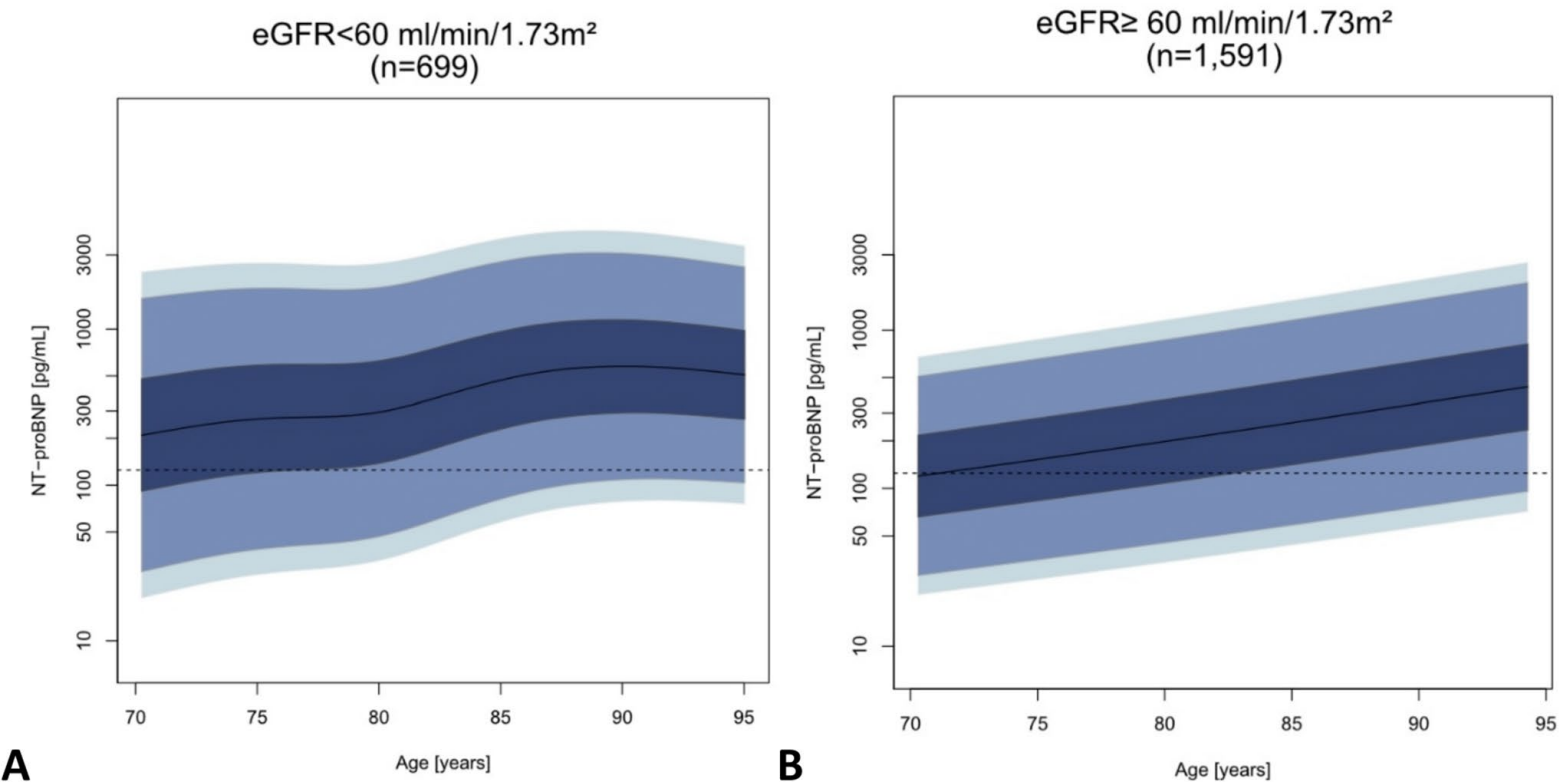
Percentile curves in the whole study cohort divided in eGFR < 60 mL/min/1.73 m^2^ (**A** *n* = 699) and eGFR ≥ 60 mL/min/1.73 m^2^ (**B** *n* = 1591). *Y*-axis shows NT-proBNP values on a log 10-based scale. Dashed line: cut-off value 125 pg/mL; light blue: 2.5–5th percentile or 95–97.5th percentile; medium blue: 5–25th percentile or 75–95th percentile; dark blue: 25–50th percentile or 50–75th percentile; solid black line: median

First, NT-proBNP values were higher in women than in men and increased with age in the 2304 participants (Fig. 2, details in Supplementary Table 1). Since NT-proBNP levels are known to depend on renal function, we also evaluated NT-proBNP levels stratified for normal and reduced eGFR (≥ vs. < 60 mL/min/1.73 m^2^, Fig. 3, details in Supplementary Table 2). In all age groups, we found that participants with reduced eGFR had higher NT-proBNP levels than participants with normal eGFR. Median NT-proBNP values were above the 125 pg/mL cut-off in men and women, in the old and very old, independent of kidney function.

### NT-proBNP distribution in the “GP-healthy” old and very old population

**Fig. 4:**
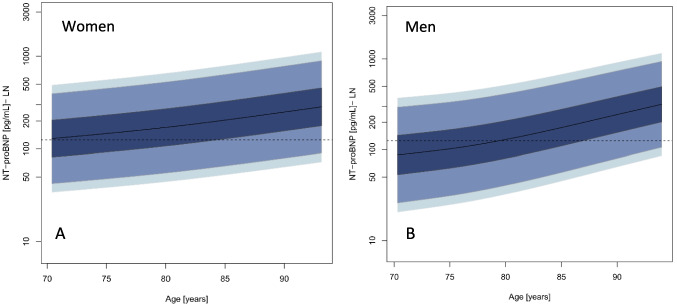
Percentile curves in “GP-healthy” subjects (subgroup II) divided in men (**B** *n* = 261) and women (**A** *n* = 388). *Y*-axis shows NT-proBNP values on a log 10-based scale. Dashed line: cut-off value 125 pg/mL; light blue: 2.5–5th percentile or 95–97.5th percentile; medium blue: 5–25th percentile or 75–95th percentile; dark blue: 25–50th percentile or 50–75th percentile; solid black line: median

Next, we analyzed the distribution of NT-proBNP in healthy subgroups. In the GP-healthy subgroup, we focused on individuals that would be deemed healthy by a routine GP check-up (no Echo exam): we analyzed the 685 participants without diabetes mellitus, obesity, elevated blood pressure, history of cardiovascular disease, kidney disease, or irregular heart rhythm. This apparently healthy subgroup showed increasing NT-proBNP by age; median levels were above 125 pg/mL across the full age range from 70 to 95 years in women, while mens’ levels were slightly lower and above 125 pg/mL at the age of 75 and older (Fig. 4A). These curves can be easily interpreted: when a 70-year-old woman is seen in practice with a value of 200 pg/mL, this would be at the 75th percentile of the healthy at this age and for this sex. For a man with 200 pg/mL, this would be at 85th percentile. Upper reference levels (97.5th percentiles) for the “GP-healthy” group ranged between 539 pg/mL in men aged 70–79 years and 825 pg/mL in women above 80 years of age (Table 2). Together, the 97.5th percentiles were derived considerably above the recommended rule-out cut-off value of 125 pg/mL.

**Table 2:** 97.5th percentiles of NT-proBNP for old (70–79 years) and very old (≥ 80 years) “GP-healthy” subjects (subgroup II). Shown are modelled 97.5th percentiles for the midpoint of each age interval resulting from GAMLSS for “GP-healthy” subjects (*n* = 649). Percentile values correspond to reference curves in Fig. 4

	Women		Men	
	70–79 years (*n* = 310)	≥ 80 years (*n* = 78)	70–79 years (*n* = 205)	≥ 80 years (*n* = 56)
NT-proBNP (pg/mL) 97.5th percentile	617.5	939.1	538.7	645.4
NT-proBNP (pg/mL) 95th percentile	468.0	825.0	360.0	588.30

### Proportion above the NT-proBNP cut-off value (125 pg/mL) for heart failure in the old and very old population

**Fig. 5:**
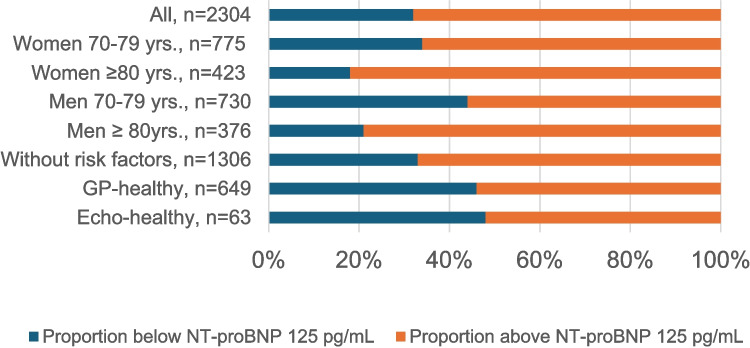
Proportion above and below the NT-proBNP cut-off value 125 pg/mL in different subgroups. Without cardiovascular risk factors: subjects free of diabetes, obesity (BMI < 30 kg/m^2^), controlled blood pressure at study visit (< 160/100 mmHg). GP-healthy: additionally with normal renal function (eGFR ≥ 60 mL/min/1.73 m^2^) and regular heart rhythm, free of previous cardiovascular disease (stent, bypass graft, myocardial infarction); no intake of high-ceiling diuretics, NOAC, or vitamin K antagonists. Echo-healthy: additionally free of left ventricular hypertrophy (left ventricular mass to body surface area ≤ 95 g/m^2^ for women/≤ 115 g/m^2^ for men) and free of left atrial enlargement (left atrial volume/body surface area ≤ 34 mL/m.^2^) with normal left ventricular filling pressure (E/e′ ratio ≤ 14) and systolic function (EF ≥ 50%)

The AHA- and ESC-guidelines endorse a rule-out cut-off value of 125 pg/mL to exclude heart failure in a non-acute setting [2, 4]. In our study, 68.1% (*n* = 1569 of 2304) of all subjects showed values above this cut-off value (Fig. 5). Individuals with NT-proBNP levels > 125 pg/mL were more likely to be women, not in regular rhythm, and suffering from left ventricular hypertrophy and coronary artery disease. Among men with NT-proBNP above the rule-out cut-off value, elevated left ventricular filling pressure (E/e′-ratio > 14) and reduced ejection fraction were more common. In women, filling pressure and ejection fraction were equally distributed among subjects below and above 125 pg/mL (Table 3).

**Table 3:** Characteristics of the study sample divided by the recommended NT-proBNP rule-out cut-off value of chronic heart failure (125 pg/mL) stratified by sex. Shown are mean and SD or proportions (if not indicated otherwise)

Women					Men			
NT-proBNP	< 125 pg/mL	*n* (339)	≥ 125 pg/mL	*n* (859)	< 125 pg/mL	*n* (396)	≥ 125 pg/mL	*n* (710)
Age (years)	77.1 ± 4.1	339	79.3 ± 5.1	859	76.5 ± 3.9	396	79.6 ± 5.4	710
BMI (kg/m^2^)	27.5 ± 4.7	338	27.6 ± 5.1	854	27.9 ± 3.9	396	27.4 ± 3.8	704
eGFR (mL/min/1.73m^2^)	74.1 ± 14.4	336	65.3 ± 16.8	855	73.7 ± 13.2	393	64.4 ± 17.4	706
LDL (mg/dL)	149.5 ± 34.1	339	146.8 ± 34.8	857	138.2 ± 33.2	396	131.0 ± 34.4	710
Inadequately controlled blood pressure (*n* (%))	15 (4.4)	339	65 (7.6)	855	28 (7.1)	396	65 (9.2)	708
Diabetes (*n* (%))	70 (20.6)	339	156 (18.2)	860	78 (19.7)	396	179 (25.2)	710
CAD (*n* (%))	6 (1.8)	333	73 (8.7)	835	40 (10.5)	380	177 (25.9)	683
Tobacco use (present/past) (*n* (%))	99 (29.3)	388	248 (29.1)	853	239 (60.4)	396	438 (62.0)	706
Regular rhythm (*n* (%))	310 (91.4)	339	671 (78.6)	854	333 (84.1)	396	450 (63.8)	705
Heart rate (bpm)	73.9 ± 10.8	339	70.0 ± 11.0	855	70.1 ± 11.6	396	67.7 ± 12.6	708
Echo-substudy								
LA-enlargement (*n* (%))	48 (42.5)	113	206 (65.8)	313	73 (39.5)	185	242 (69.1)	350
LV-Hypertrophy (*n* (%))	34 (34.7)	98	132 (49.4)	267	56 (40.0)	140	147 (53.1)	277
E/e′ ratio > 14 (*n* (%))	21 (19.6)	107	62 (21.6)	287	19 (11.0)	172	68 (21.9)	311
LVEF < 50% (*n* (%))	7 (6.0)	117	19 (5.9)	323	14 (7.6)	185	51 (14.5)	351

*n* with available data

*BMI* body mass index, *eGFR* glomerular filtration rate estimated from serum creatinine (mL/min/1.73m^2^); inadequately controlled blood pressure: RR > 160/100 mmHg at study visit; *CAD* coronary artery disease: myocardial infarction, stents, coronary artery bypass grafting; left atrial enlargement: left atrial volume/body surface area > 34 mL/m^2^; LV hypertrophy: left ventricular mass to body surface area > 95 g/m^2^ for women, > 115 g/m^2^ for men; *LVEF* left ventricular ejection fraction

The proportion above the rule-out cut-off value in cardiovascular healthy individuals can provide an estimate for the proportion of false-positives when applying the rule-out value without a critical look based on the age of the individual.

First, in a subgroup without cardiovascular risk factors (free of diabetes mellitus and obesity with controlled blood pressure), 67.2% of the participants still showed values above 125 pg/mL. In the “GP-healthy”-subgroup (*n* = 649), in which participants with a history of cardiovascular disease, renal failure, intake of high-ceiling diuretics, or irregular heart rhythm were additionally excluded, 49% (70 to 79 years) and 69% (> 80 years) were above the rule-out cut-off value. Finally, in the “echo-healthy”-subgroup (*n* = 63), which is additionally free of left ventricular hypertrophy and diastolic or systolic dysfunction, 52% of the subjects were determined to have false-positive results for heart failure based on NT-proBNP (Fig. 5).

Our results indicate that the probability of being wrongly identified as potential heart failure patient is more than 50% for older individuals, particularly for women, by applying the guideline recommended rule-out level of 125 pg/mL.

### Factors independently influencing NT-proBNP

After adjusting for sex and age, several parameters were analyzed separately in a univariate linear regression model (Supplementary Table 3). Factors that showed a significant effect were subsequently included in a multivariate linear regression model. In our study, lower eGFR, irregular heart rhythm, larger left atrial volume/body surface area, lower EF, higher E/e′ and intake of NOAC, vitamin K antagonists, or beta-blockers were independent factors for higher NT-proBNP levels. Higher BMI was associated with lower NT-proBNP values (Table 4).

**Table 4:** Factors independently influencing NT-proBNP

lnNT-proBNP	*β*	95% CI	*p*-value	*R*^2^	*n*
Sex (0-f; 1-m)	− 0.19	− 0.33; − 0.05	0.007	0.436	637
Age (y)	0.04	0.02; 0.05	3.4 × 10^−7^		
eGFR (mL/min/1.73 m^2^)	− 0.01	− 0.02; − 0.01	1.8 × 10^−7^		
NOAC (0-no; 1-yes)	0.33	0.12; 0.63	0.034		
Vitamin K antagonist (0-no; 1-yes)	0.37	0.09; 0.64	0.009		
High ceiling diuretics (0-no; 1-yes)	0.19	− 0.03; 0.40	0.091		
Coronary artery disease (0-no; 1-yes)	0.16	− 0.03; 0.34	0.090		
Arterial hypertension (0-no; 1-yes)	− 0.10	− 0.25; 0.04	0.168		
Beta blockers (0-no; 1-yes)	0.32	0.18; 0.46	8.2 × 10^−6^		
Regular heart rhythm (1- no; 2-yes)	− 0.65	− 0.91; − 0.40	8.0 × 10^−7^		
LAVI (mL/m^2^)	0.02	0.01; 0.02	3.1 × 10^−9^		
LVMi (g/m^2^)	0.00	− 0.00; 0.00	0.447		
EF (%)	− 0.02	− 0.03; − 0.01	4.2 × 10^−5^		
E/e′ ratio	0.02	0.00; 0.04	0.018		

multivariate analysis of lnNT-proBNP

*eGFR* glomerular filtrations rate estimated from serum creatinine using CKD-Epi equation (mL/min/1.73 m^2^); NOAK: direct oral anticoagulants; arterial hypertension: resting systolic blood pressure > 140 mmHg and/or diastolic blood pressure > 90 mmHg and/or current antihypertensive drug therapy; coronary artery disease: self-reported myocardial infarction, stents, coronary artery bypass grafting; *LAVI* left atrial volume index (left atrial volume/body surface area), *LVMi* ratio of left ventricular mass to body surface area, *LVEF* left ventricular ejection fraction; E/e′ ratio: mitral valve E velocity divided by mitral annular e′ velocity

## Discussion

In our study sample of 2304 community-dwelling, old and very old participants, NT-proBNP levels continue to rise with age, even among 685 seemingly healthy subjects of the “GP-healthy” subgroup. The 97.5th percentiles of GP-healthy subjects are considerably higher than the recommended exclusion cut-off level for the ambulatory setting across all age groups in women and men. Our results from the AugUR study indicate that the established, age-independent rule-out cut-off value of > 125 pg/mL for chronic heart failure results in a high proportion of false positives among individuals aged 70 years and older.

BNP exerts cardiac, renal, vascular, and muscular effects [21–24]. The more stable NT-proBNP is mostly used as a biomarker. Though cardiac wall stretch is the dominant stimulus for secretion, NT-proBNP serum levels depend on further variables, rendering interpretation in the clinical context difficult.

Women have higher NT-proBNP values than men in the general adult population [5, 25–27]. Age dependency and sex dependency have been reported in the younger population for both BNP [25] and MR-proANP [28]. We found this also in our study among individuals at 70, 80, or even 90 years of age. Both sex hormones were suspected to influence natriuretic peptide levels—testosterone may reduce, while oestrogen may increase natriuretic peptide levels [29, 30]. However, further research is needed to determine why this sex dependency persisted in old and very old people. In our study of an elderly population, the factors associated with elevated NT-proBNP were consistent with those identified in previous studies involving younger cohorts [26, 31].

Early data on age-dependency of NT-proBNP levels were derived from the Olmsted County and Copenhagen studies [25, 26, 32], but the number of older participants was low (Olmsted County study 275 participants > 75 years, Copenhagen 294 participants > 70 years). In the meantime, additional population-based samples have validated age-dependency in general adults. However, even the largest of these studies, the Generation Scotland Scottish Family Healthy Study (GS:SFHS), included only 710 participants aged 70 years or older. Thus, 3.3% of 18,356 individuals in GS:SFHS were 70 years or older, despite 19% of Scots being of pension age [33]. As in GS:SFHS, higher age groups are underrepresented in most population-based samples.

Due to the small numbers of old and very old participants in previous studies, valid age-dependent thresholds have not been established yet for NT-proBNP to diagnose chronic heart failure, whereas in acute heart failure, age-dependent reference values have already been recommended [10, 25].

A joint analysis of ActiFE Ulm and SHIP focused on individuals aged at least 65 years and defined an apparently healthy subgroup, as NT-proBNP levels depend on different cardiac diseases, renal function [34], and obesity [5]. However, only 220 of them were in an age of 70 years or older, not providing reliable reference values in necessary strata (at least sex). The need for age-appropriate thresholds is urging, as older patients are the primary target population of NT-proBNP testing. The prevalence of heart failure increases with age, with up to fourfold higher prevalence (8.0–9.1%) among US adults above 65 years than with age less than 65 [35]. The Heart failure guidelines of the AHA consider the measurement of NT-proBNP in patients presenting with dyspnea as useful to support a diagnosis or exclusion of heart failure (Class of recommendation 1, level of evidence A) [4]. European guidelines on heart failure diagnosis recommend NT-proBNP testing in all patients with suspected chronic heart failure (Class I, Level of evidence B) [2]. Hence, about 70% of all NT-proBNP measurements are done in patients aged at least 75 years [9]. Strict obedience to the guidelines recommended rule-out cut-off value of 125 pg/mL leads to unnecessary referrals of about 74% of patients aged 80 years and older to cardiologists [8].

The lack of thresholds and the high rate of false-positive results tie up more healthcare resources than necessary, increase costs and patients’ concerns, and lead to unnecessary transports of less mobile patients. It is important to understand what is a normal NT-proBNP level in the older population and what its reason for concern is to prompt referral. As NT-proBNP levels are associated with cardiac diseases, thorough cardiac phenotyping of the study sample is necessary to define reference values in a healthy subgroup. For instance, tachycardia increases workload and leads to heart failure [36–38]. However, data on heart rhythm are often lacking in population-based studies like ActiFE Ulm and SHIP [5]. In our “GP-healthy” subgroup, we excluded all individuals with irregular heart rhythm. Additionally, NT-proBNP is associated with even subtle remodeling of cardiac morphology and function. Echocardiography reveals these processes and facilitates an “echo-healthy” subgroup in our analyses. In population-based studies, echocardiography is often subjected to resource constraints due to the expensive equipment and the prerequisite of trained sonographers. Thus, echocardiographic phenotyping was lacking in most previous studies on NT-proBNP in the older population (e.g., in GS:SFHS, ActiFE, getABI [5, 39, 40]). On the other hand, some limitations of echocardiography in older adults should be considered. Like the NT-proBNP cut-off level, most echocardiographic reference values are based on a younger population [18], and age-specific reference values are lacking. Thus, the “echo-healthy” subgroup might be in a very good cardiac condition beyond the average of peers of the same age.

Further data is certainly needed for refinements of the current cut-off levels. To overcome the constraints of cut-off levels, artificial intelligence-based approaches have been proposed for the interpretation of natriuretic peptides [41, 42]. We provide reference data on which AI might be trained for old and very old patients.

Considering the small study samples of ours and others, forthcoming meta-analyses may set further reference values of NT-proBNP.

### Study limitations

The recruitment strategy of our study led to a selection towards healthier subjects. The participants’ mental and physical health had to be sufficient to travel on their own to the study center and to answer all interview questions. However, this selection bias does not limit but supports the aims of our analysis, as we set out to define reference distributions of NT-proBNP in a healthy older population. GP healthy subgroup differs in their selection criteria from IFCC criteria (e.g., detailed medication history like lipid-lowering agents, antihypertensives, history of pre-existing conditions like pulmonary disease, cancer).

A major limitation for the clinical translation of our data is that we did not validate our population-based reference values in a heart failure cohort, which is the aim of future studies.

Elevated NT-proBNP levels are associated with the prevalence of atrial fibrillation [43]. Our study excluded subjects with arrhythmias documented at the study site from the healthy subgroups (“GP-healthy,” “echo-healthy”). As we did not record Holter monitor electrocardiograms, we cannot exclude individuals with previously undiagnosed paroxysmal atrial fibrillation not present during the study visit. Even if this small subgroup could contribute to elevated NT-proBNP levels, the number of concerned individuals is rather low, with a prevalence of less than 3.5% in the older population [44], and they might be already largely excluded due to the very high correlation of atrial fibrillation and other diseases, which we asked for (e.g., hypertension), measured (e.g., glomerular filtration rate), or analyzed by echocardiography (e.g., left ventricular hypertrophy).

## Conclusions

NT-proBNP levels continue to increase with age in the old and very old adults, which remains true for subjects free of known cardiac diseases and with normal cardiac morphology and function. Furthermore, women show higher NT-proBNP values than men even in advanced, postmenopausal age. Applying the guideline-recommended exclusion cut-off values, the resulting high false-positive rate may contribute to an unnecessary increase in patients’ concerns and healthcare costs. Our reference value curves in cardiovascularly healthy old and very old individuals can help practitioners to put observed NT-proBNP values of old aged individuals into perspective.

### Clinical perspectives

Competency in patient care: In older patients, higher NT-proBNP levels may be assumed, even in the absence of heart failure. Beyond NT-proBNP measurement, clinical reasoning on dyspnoeic older patients has to take into account further anamnestic and diagnostic information and may less rely on age-independent cut-off values.

Translational outlook: The guideline endorsed age- and sex-independent rule-out cut-off values of NT-proBNP imply low specificity among older individuals. Our study may contribute epidemiologic data for further meta-analyses of NT-proBNP thresholds in the older population, in which the prevalence of heart failure is highest, whereas population-based cut-off values are based on a more tenuous database compared to younger individuals.

## Supplementary Information

Below is the link to the electronic supplementary material.

Supplementary file1 (DOCX 39 KB)

## Data Availability

The datasets generated and analysed during the current study are not publicly available due to data privacy of study participants, but summary data are available from the corresponding authors on reasonable request.
