# Supplementary material for: NT-proBNP in the very old population: reference values and heart failure specificity from the AugUR-study
Source: Clin Res Cardiol. 2025 Oct 27;115(9):1571–82. doi: 10.1007/s00392-025-02785-3 (PMC13457255; doi:10.1007/s00392-025-02785-3)
Supplement: Supplementary file 1 [file 392_2025_2785_MOESM1_ESM.docx]

**Supplementary**

|  | | | n | Median (P25; P75) | Proportion above 125 pg/mL [%] |
| --- | --- | --- | --- | --- | --- |
| All |  |  | 2304 | 187.0 (106.0; 380.0) | 68.1 |
|  | stratified by sex | women | 1198 | 195.0 (116.0;376.0) | 71.7 |
|  |  | men | 1106 | 179.7 (96.2; 396.0) | 64.2 |
|  | stratified by sex and age | women 70-79 years | 775 | 166.6 (103.0; 306.3) | 66.3 |
|  |  | women 80-95 years | 423 | 262.4 (157.4; 547.5) | 81.6 |
|  |  | men 70-79 years | 730 | 148.0 (80.8; 282.0) | 56.4 |
|  |  | men 80-95 years | 376 | 295.5 (134.9; 706.5) | 79.3 |
|  | stratified by kidney function | eGFR ≥ 60 | 1591 | 162.0 (95.6; 303.0) | 61.5 |
|  |  | eGFR <60 | 699 | 286.0 (150.0;729.0) | 83.3 |
| Subgroup I |  |  | 1306 | 180.8 (104.4; 355.0) | 67.2 |
|  | stratified by sex | women | 690 | 192.2 (116.0; 358.5) | 71.4 |
|  |  | men | 616 | 170.2 (93.4; 350.5) | 62.3 |
|  | stratified by age groups | 70-79 years | 880 | 154.0 (93.1;269.9) | 60.2 |
|  |  | ≥ 80 years | 426 | 273.4 (150.0; 556.0) | 81.5 |
| Subgroup II |  |  | 649 | 131.1 (86.3; 214.8) | 53.5 |
|  | stratified by sex | women | 388 | 152.5 (95.9;235.5) | 61.1 |
|  |  | men | 261 | 107.8 (70.8; 186.0) | 42.1 |
|  | stratified by age groups | 70-79 years | 515 | 123.2 (97.6;198.0) | 49.3 |
|  |  | ≥ 80 years | 134 | 188.5 (109.2; 295.0) | 69.4 |
| Subgroup III |  |  | 63 | 133.6 (79.7; 194.8) | 52.4 |
|  | stratified by sex | women | 35 | 173.9 (92.8;244.5) | 65.7 |
|  |  | men | 28 | 91.2 (69.8;158.9) | 35.7 |
|  | stratified by age group | 70-79 years | 60 | 138.1 (81.5;196.5) | 51.7 |
|  |  | ≥ 80 years | 3 | 133.6 (71.2;157.4) | 66.7 |

**Supplementary Table 1: Distribution of NT-proBNP values and percentiles corresponding to the recommended rule-out cut-off value for heart failure diagnosis (125pg/mL) in subgroups free of cardiovascular risk factors, diseases and with normal left ventricular morphology and function.** Shown are median, 25th and 75th percentiles in the entire AugUR study sample (all) with further stratification for sex, age and renal function, as well as subgroup I without cardiovascular risk factors, the “GP-healthy” subgroup II and the “echo-healthy” subgroup III.

*Subgroup I: “Without cardiovascular risk factors”: subjects free of diabetes, obesity (BMI <30 kg/m^2^), controlled blood pressure at study visit (< 160/100 mmHg)*

*Subgroup II: “GP-healthy”: subgroup I and additionally with normal renal function (eGFR ≥ 60 mL/min/1.73 m^2^) and regular heart rhythm, free of previous cardiovascular disease (stent, bypass graft, myocardial infarction); no intake of high ceiling diuretics, NOACs or vitamin K antagonists.*

*Subgroup III: “Echo-healthy”: subgroup II and additionally free of left ventricular hypertrophy (left ventricular mass to body surface area ≤ 95 g/m^2^ for women/ ≤ 115 g/m^2^ for men) and free of left atrial enlargement (left atrial volume/body surface area ≤ 34 mL/m^2^) with normal left ventricular filling pressure (E/e’ ratio ≤ 14) and systolic function (EF ≥ 50%).*

**Supplementary Table 2: NT-proBNP values [pg/mL] by age groups and eGFR [mL/min/1.73 m^2^] in 2,290 participants of the AugUR study**

| Age groups | 70-74 yrs. | 75-79 yrs. | 80-84 yrs. | 85-89 yrs. | >90 yrs. | All |
| --- | --- | --- | --- | --- | --- | --- |
| eGFR ≥ 60 [mL/min/1.73m^2^], n | 519 | 626 | 328 | 94 | 24 | 1591 |
| 5^th^ percentile | 34.7 | 44.9 | 61.4 | 62.3 | 145.2 | 43.4 |
| 10^th^ percentile | 47.1 | 61.3 | 69.9 | 81.5 | 154.6 | 59.5 |
| 25^th^ percentile | 81.1 | 92.8 | 109.4 | 158.0 | 237.5 | 95.6 |
| median | 131.0 | 155.0 | 197.5 | 297.3 | 396.0 | 162.0 |
| 75^th^ percentile | 232.0 | 278.0 | 379.1 | 576.0 | 694.5 | 303.0 |
| 90^th^ percentile | 467.2 | 534.9 | 720.8 | 1427.5 | 1162.0 | 616.5 |
| 95^th^ percentile | 814.0 | 938.6 | 1190.0 | 1547.0 | 1226.0 | 1013.0 |
| eGFR < 60 [mL/min/1.73m^2^], n | 105 | 244 | 191 | 122 | 37 | 699 |
| 5^th^ percentile | 46.2 | 52.1 | 72.1 | 119.0 | 128.0 | 58.0 |
| 10^th^ percentile | 54.5 | 71.0 | 101.4 | 151.4 | 164.6 | 89.7 |
| 25^th^ percentile | 105.0 | 140.9 | 147.5 | 244.0 | 311.9 | 150.0 |
| median | 192.3 | 241.5 | 323.4 | 507.8 | 431.3 | 286.0 |
| 75^th^ percentile | 469.7 | 482.0 | 729.0 | 1249.0 | 1217.0 | 729.0 |
| 90^th^ percentile | 1701.4 | 1503.5 | 1877.2 | 2227.6 | 1829.6 | 1785.0 |
| 95^th^ percentile | 2355.0 | 2346.0 | 2524.0 | 2784.0 | 3690.0 | 2524.0 |

*eGFR: glomerular filtrations rate estimated from serum creatinine using* CKD-Epi equation *[mL/min/1.73 m^2^]*

**Supplementary Table 3: Univariate linear regression model for lnNTproBNP**

| Ln NT-proBNP | **β** | **95% CI** | **p-value** | **R2** | **n** |
| --- | --- | --- | --- | --- | --- |
| Sex [0-m; 1-f] | -0.42 | -0.12;0.04 | 0.289 | 0.172 | 2286 |
| Age [y] | 0.05 | 0.04;0.06 | 8.5*10^-30^ |  |  |
| eGFR | -0.18 | -0.02;-0.01 | 1.5*10^-43^ |  |  |

| Ln NT-proBNP | **β** | **95% CI** | **p-value** | **R2** | **n** |
| --- | --- | --- | --- | --- | --- |
| Sex [0-m; 1-f] | -0.08 | -0.16;-0.00 | 0.044 | 0.191 | 2296 |
| Age [y] | 0.06 | 0.05;0.07 | 9.2*10^-53^ |  |  |
| Vitamin K antagonist [0-no; 1-yes] | 1.27 | 1.11;1.42 | 5.6*10^-55^ |  |  |
| Ln NT-proBNP | **β** | **95% CI** | **p-value** | **R2** | **n** |
| Sex [0-m; 1-f] | -0.04 | -0.11; 0.04 | 0.355 | 0.223 | 2296 |
| Age [y] | 0.06 | 0.05;0.07 | 4.1*10^-51^ |  |  |
| Betablocker [0-no; 1-yes] | 0.76 | 0.68;0.83 | 5.4*10^-75^ |  |  |

| Ln NT-proBNP | **β** | **95% CI** | **p-value** | **R2** | **n** |
| --- | --- | --- | --- | --- | --- |
| Sex [0-m; 1-f] | -0.15 | -0.26;-0.03 | 0.012 | 0.294 | 1004 |
| Age [y] | 0.05 | 0.05;0.07 | 5.8*10^-26^ |  |  |
| Regular rhythm [1-no; 2-yes] | -1.44 | -1.61;-1.26 | 5.4*10^-51^ |  |  |

| Ln NT-proBNP | **β** | **95% CI** | **p-value** | **R2** | **n** |
| --- | --- | --- | --- | --- | --- |
| Sex [0-m; 1-f] | -0.04 | -0.18;0.09 | 0.545 | 0.144 | 873 |
| Age [y] | 0.07 | 0.05;0.08 | 3.3*10^-20^ |  |  |
| E/e´ | 0.05 | 0.03;0.07 | 2.6*10^-7^ |  |  |

| Ln NT-proBNP | **β** | **95% CI** | **p-value** | **R2** | **n** |
| --- | --- | --- | --- | --- | --- |
| Sex [0-m; 1-f] | -0.19 | -0.34;-0.03 | 0.018 | 0.124 | 778 |
| Age [y] | 0.07 | 0.05;0.08 | 1.6*10^-17^ |  |  |
| LV mass index (g/m²) | 0.01 | 0.00;0.01 | 1.7*10^-6^ |  |  |

| Ln NT-proBNP | **β** | **95% CI** | **p-value** | **R2** | **n** |
| --- | --- | --- | --- | --- | --- |
| Sex [0-m; 1-f] | -0.06 | -0.18;0.06 | 0.302 | 0.300 | 957 |
| Age [y] | 0.06 | 0.04;0.07 | 5.1*10^-19^ |  |  |
| LA vol index(ml/m²) | 0.03 | 0.03;0.04 | 2.5*10^-49^ |  |  |

| Ln NT-proBNP | **β** | **95% CI** | **p-value** | **R2** | **n** |
| --- | --- | --- | --- | --- | --- |
| Sex [0-m; 1-f] | -0.16 | -0.29;- 0.04 | 0.011 | 0.177 | 972 |
| Age [y] | 0.08 | 0.06;0.09 | 1.2*10^-30^ |  |  |
| LVEF (%) | -0.04 | -0.04;-0.03 | 5.6*10^-16^ |  |  |

| Ln NT-proBNP | **β** | **95% CI** | **p-value** | **R2** | **n** |
| --- | --- | --- | --- | --- | --- |
| Sex [0-m; 1-f] | -0.12 | -0.20;-0.03 | 0.006 | 0.136 | 2227 |
| Age [y] | 0.06 | 0.05;0.07 | 2.0*10^-47^ |  |  |
| CVD | -0.61 | -0.49;0.74 | 3.5*10^-22^ |  |  |

| Ln NT-proBNP | **β** | **95% CI** | **p-value** | **R2** | **n** |
| --- | --- | --- | --- | --- | --- |
| Sex [0-m; 1-f] | -0.04 | -0.12;0.04 | 0.302 | 0.109 | 2296 |
| Age [y] | 0.06 | 0.06;0.07 | 2.1*10^-52^ |  |  |
| Hypertension | 0.22 | 0.13;0.32 | 1.5*10^-5^ |  |  |

| Ln NT-proBNP | **β** | **95% CI** | **p-value** | **R2** | **n** |
| --- | --- | --- | --- | --- | --- |
| Sex [0-m; 1-f] | -0.08 | -0.15;0.00 | 0.055 | 0.202 | 2296 |
| Age [y] | 0.06 | 0.05;0.06 | 2.6*10^-44^ |  |  |
| High ceilng diuretics [0-no; 1-yes] | 1.00 | 0.88;1.11 | 1.9*10^-61^ |  |  |
